# Obstructive Sleep Apnea Disrupts Glycemic Control in Obese Individuals

**DOI:** 10.3390/medicina58111602

**Published:** 2022-11-05

**Authors:** Christopher Seifen, Johannes Pordzik, Katharina Ludwig, Katharina Bahr, Cornelia Schupp, Christoph Matthias, Perikles Simon, Haralampos Gouveris

**Affiliations:** 1 Sleep Medicine Center & Department of Otolaryngology, Head and Neck Surgery, University Medical Center Mainz, 55131 Mainz, Germany; 2 Department of Sports Medicine, Disease Prevention and Rehabilitation, Johannes Gutenberg University, 55099 Mainz, Germany

**Keywords:** obstructive sleep apnea, apnea–hypopnea index, body mass index, obesity and obstructive sleep apnea, HbA1c, CRP

## Abstract

The link between obstructive sleep apnea (OSA) and obesity, and their common comorbidities such as diabetes mellitus (DM) or cardiovascular diseases, is not fully understood. The aim of this study was to investigate the possible association of OSA severity in obese individuals with polysomnography-based sleep parameters, as well as C-reactive protein (CRP) and glycated hemoglobin (HbA1c) serum levels. Polysomnographic recordings and blood samples were retrospectively compared between a group of 23 adult obese individuals with mild OSA (apnea–hypopnea index (AHI) = 7.5 (5.5–12.5)/h, age = 42.57 ± 11.44 years, 16 male, 7 female, body mass index (BMI) = 37.35 ± 3.88 kg/m^2^) and an age-, sex-, and BMI-matched group of individuals with moderate or severe OSA (AHI 41.5 (25.7–71.8)/h, age = 43.43 ± 11.96 years, 16 male, 7 female, BMI = 37.87 ± 4.74 kg/m^2^). All respiratory sleep-associated parameters were significantly higher in individuals with moderate and severe OSA compared to those with mild OSA. CRP levels did not differ between the two OSA severity groups. However, serum levels of HbA1c were significantly higher in the moderate/severe OSA group. Therefore, OSA severity may have a significant impact on glycemic control in obese individuals. Additionally, OSA severity did not appear to be further associated with systemic inflammation in obese individuals. Obese individuals may benefit not only from lifestyle modification, but also from OSA screening and treatment, particularly to prevent DM-associated disorders and conditions.

## 1. Introduction

Obstructive sleep apnea (OSA) is the most common type of sleep-disordered breathing. OSA is characterized by recurrent episodes of partial or complete airway obstruction during sleep, leading to repetitive apneas or hypopneas. Airway obstruction results from upper airway collapse or anatomic airway obstruction, even though respiratory effort is still present [1]. The strongest risk factor for developing OSA is being overweight, and in particular, elevated body mass index (BMI) [2]. Recent estimates suggest that 60% of the adult population in industrialized countries is overweight (BMI ≥ 25 kg/m^2^) and at least 30% is obese (BMI ≥ 30 kg/m^2^) [3]. In obese individuals, the airway is narrowed by fat deposits in the upper respiratory tract and muscle activity in this region is decreased, both leading to apneic and hypoxic episodes. It is estimated that 58% of moderate to severe OSA is due to obesity [4,5,6].

Both OSA and obesity have been correlated with the development of metabolic syndrome and cardio-metabolic diseases such as systemic hypertension and diabetes mellitus (DM) [7,8]. With regards to DM, it is suggested that 15–30% of individuals with OSA have type 2 diabetes mellitus (T2DM), and that OSA is associated with insulin resistance and glucose intolerance [9]. A possible link between OSA and the development of T2DM may be the result of sympathetic nervous system activation by intermittent hypoxia and sleep fragmentation, or insulin resistance resulting from the increased release of inflammatory cytokines [10].

Additionally, reports in the literature suggest that OSA might be responsible for low-level systemic inflammation and subsequent vascular damage [11,12]. This condition has been suggested to be the underlying mechanism responsible for numerous comorbidities, e.g., cardiovascular diseases and DM [13]. Thus, the detection of altered inflammatory markers in individuals with OSA may predict the degree of nocturnal sleep disturbance and associated systemic inflammation.

As a matter of fact, not all obese individuals show moderate to severe OSA and not all individuals with OSA develop impaired glucose metabolism or systemic inflammation. Therefore, the aim of this study was to compare sleep-related and biochemical markers between two groups of obese sex-, age-, and BMI-matched individuals—one with mild and one with moderate or severe OSA. We hypothesized that polysomnographic parameters would differ significantly in obese individuals with moderate/severe OSA compared to those with mild OSA. Additionally, specific attention was paid to the potential role of inflammatory serum marker C-reactive protein (CRP) and glycated hemoglobin (HbA1c) levels as pivotal markers for systemic glycemic control.

## 2. Materials and Methods

### 2.1. Study Protocol

The clinical database of our sleep laboratory (of a tertiary university medical center) was searched retrospectively between January 2019 and June 2022 for individuals aged over 18 years old with a BMI greater than 32 kg/m^2^ undergoing polysomnography according to the American Academy of Sleep Medicine (AASM) standard guidelines. Individuals under 18 years of age, as well as individuals with positive medical history of heart attack, anemia, end-stage renal disease, recent exacerbation of asthma or chronic obstructive pulmonary disease (COPD), recent infection or severe chronic mental health disorders (e.g., schizophrenia, and psychosis) were excluded from the study. In order to assess the severity of sleep apnea, the apnea–hypopnea index (AHI) was used. The AHI was calculated as the sum of the total number of obstructive, mixed and central apneas, as well as hypopneas occurring per hour of sleep. An AHI of 5 to <15/h is classified as mild, ≥15 but <30/h is considered moderate and >30/h is considered severe [14]. 

The baseline evaluation of all individuals included assessment of clinical and demographic characteristics including age, sex, and BMI. Medical records of all individuals were reviewed to search for medical conditions and permanent medication. Two groups were formed: Individuals with BMI >32 kg/m^2^ and AHI < 15/h were assigned to group “AHI < 15”, while individuals with BMI >32 kg/m^2^ and AHI ≥ 15/h were assigned to group “AHI ≥ 15”. Matching of individuals in both groups was performed considering age (±5 years), sex and BMI (±10 kg/m^2^). For further investigation, the polysomnography of each individual was analyzed for the following parameters: AHI; total number of apneic events per hour (apnea index), total number of hypopnea events per hour (hypopnea index); total number of snoring events per hour (snoring index); total number of oxygen desaturation events (≥4%) per hour (oxygen desaturation index); total number of oxygen desaturation events during rapid eye movement (REM) sleep; average oxygen desaturation in percentage; percentage of oxygen desaturation lower than 90% (t90); total number of periodic limb movements (PLM); total sleep time in minutes (TST); percentage of N3 sleep (representing stable breathing pattern); percentage of REM sleep (representing irregular breathing pattern); AHI during REM sleep (REM AHI); total number of arousal events per hour (arousal index); total number of hourly cortical arousals following apnea or hypopnea (respiratory arousal index); and AHI in supine position. Additionally, blood samples which were taken as part of the routine clinical practice on the day of inpatient admission, were screened for CRP and HbA1c levels.

### 2.2. Statistical Analysis 

All data were statistically analyzed using JMP 14 (SAS Institute, Cary, NC, USA). Categorical variables were described as number and percentage (%), and continuous variables were described as mean ± standard deviation for normal distributed or median and interquartile range (IQR), for non-normal distributed values. Within the figures, median and IQR are given, and individuals are visualized using dots and lines for their matching partners. Solitaire points represent individuals without matching partners or missing blood sample values. All statistical tests were performed after evaluating the normality of distribution with the Shapiro–Wilk test and Kolmogorov–Smirnov test. Comparisons between groups were analyzed using the paired *t* test for normal distributed values, and the Wilcoxon rank-sum test for non-normal distributed values. The results were considered significant when the *p*-value was < 0.05.

## 3. Results

### 3.1. Study Population

Between January 2019 and June 2022, a total of 683 individual polysomnographic records were performed. A total of 23 individuals (7 male, 16 female) met the inclusion criteria for group “AHI < 15” (BMI >32 kg/m^2^, AHI < 15/h). Age distribution in this group was 26–75 years (42.57 ± 11.44 years) and BMI was 32–46 kg/m^2^ (37.35 ± 3.88 kg/m^2^). An additional 23 individuals meeting the criteria for group “AHI ≥ 15” (BMI > 32 kg/m^2^, AHI ≥ 15/h) were recruited, and individually matched with group “AHI < 15” considering age (±5 years), sex and BMI (±10 kg/m^2^). Age distribution in group “AHI ≥ 15” was 25–73 years (43.43 ± 11.96 years) and BMI was 32–50 kg/m^2^ (37.87 ± 4.74 kg/m^2^). Basic anthropomorphic data on hypertension, diabetes mellitus, pulmonary disorders (asthma, COPD), and hypothyroidism were collected from individuals’ medical records. In addition, the medical files were examined for Insulin-, Metformin-, or Levothyroxine intake, as well as smoking status (none vs. active). Baseline characteristics of the study population are shown in Table 1.

### 3.2. Comparison of Polysomnographic Sleep Parameters

The primary goal of this study was to compare sleep parameters in obese individuals with mild OSA (group “AHI < 15”) to those with moderate to severe OSA (group “AHI ≥15”). All respiratory parameters, such as AHI, apnea index, hypopnea index and snoring index, were significantly higher in group “AHI ≥ 15” compared to group “AHI < 15” (*p* < 0.001, *p* < 0.001, *p* < 0.001 and *p* = 0.01, respectively). Evaluation of pulse oximetry measurements found oxygen desaturation index and total number of oxygen desaturation events during REM sleep to be significantly higher in obese patients with moderate to severe OSA (*p* < 0.001 and *p* < 0.001, respectively), while average oxygen desaturation and percentage of oxygen desaturation lower than 90% was significantly lower (*p* < 0.001 and *p* < 0.001, respectively). PLM were registered significantly more frequently in group “AHI ≥ 15” (*p* = 0.014). Obese people with mild OSA were found to have a significantly lower arousal index (*p* < 0.001) compared to those with moderate to severe OSA. TST and the distribution of sleep stages, more precisely the percentages of N3 and REM sleep, did not differ significantly between groups (*p* = 0.312, *p* = 0.39 and *p* = 0.746, respectively). Table 2 presents the comparison of all the aforementioned sleep parameters.

### 3.3. Analysis of C-Reactive Protein and Glycated Hemoglobin Levels

**Figure 1:**
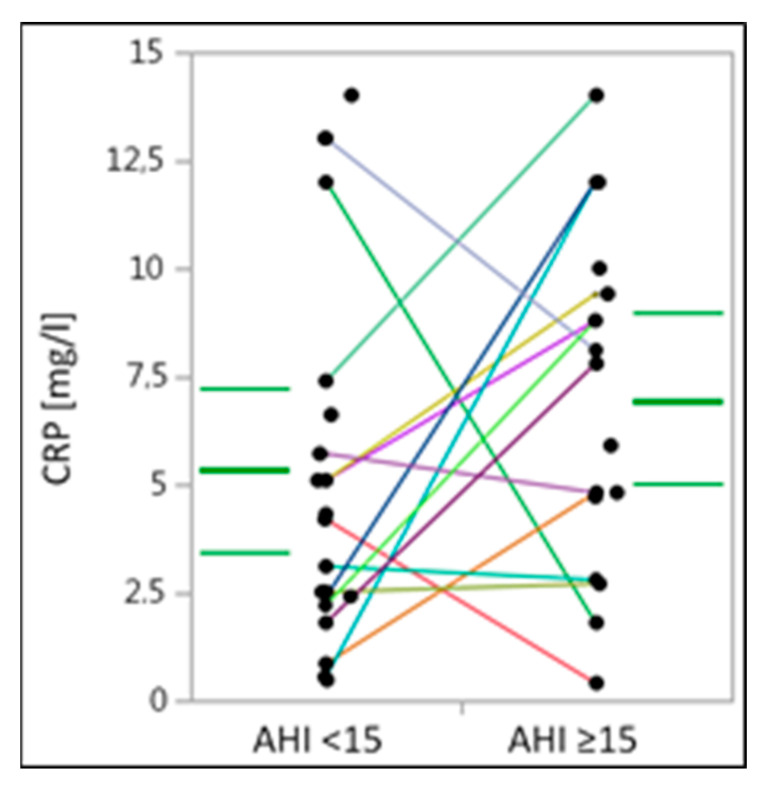
C-reactive protein levels in obese individuals with mild or moderate to severe obstructive sleep apnea. CRP-C-reactive protein. CRP-levels are presented in milligrams per liter. Within the figure, median and IQR are given (bold and thin green lines, respectively) and individuals are visualized using dots and lines for their matching partners. Solitaire points represent individuals without matching partners or missing blood sample values.

Analysis of the serum levels of the inflammatory serum marker CRP did not reveal any significant differences between obese individuals with mild OSA compared to those with moderate to severe OSA (*p* = 0.186) (Table 3) (Figure 1). However, comparing the median and IQR, group “AHI ≥ 15” showed a higher tendency toward CRP levels compared to group “AHI < 15”.

Medical record evaluation revealed two diabetic individuals in each group. Each of these individuals was treated with Metformin. One additional individual in group “AHI < 15” was taking Metformin without DM being described. Insulin therapy was not performed by any individual in the study cohort. 

**Figure 2:**
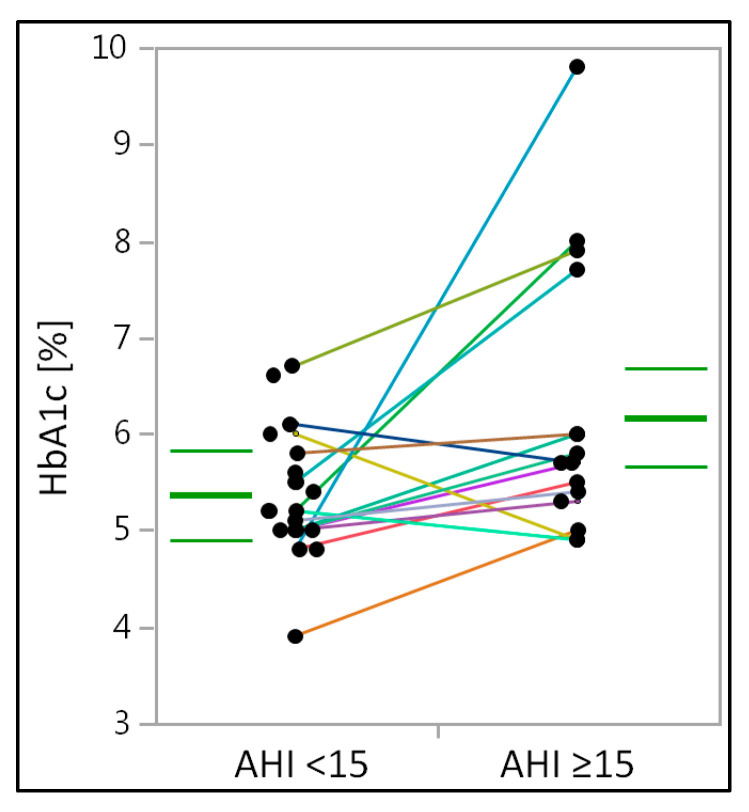
Glycated hemoglobin in obese individuals with mild or moderate to severe obstructive sleep apnea. HbA1c—glycated hemoglobin. HbA1c—levels are presented in percentages. Within the figure, median and IQR are given (bold and thin green lines, respectively) and individuals are visualized using dots and lines for their matching partners. Solitaire points represent individuals without matching partners or missing blood sample values.

However, serum levels of HbA1c, a marker for glycemic control, were significantly higher in obese individuals with moderate to severe OSA (*p* = 0.016) (Table 3) (Figure 2). Between groups, the significance of the CRP and HbA1c-levels did not change, regardless of total group comparison or paired testing.

## 4. Discussion

In this study, we demonstrated that all respiratory-related polysomnographic sleep parameters are significantly higher in age-, sex-, and BMI-matched obese individuals with moderate and severe OSA, compared to those with mild OSA. Accordingly, there was a significantly higher PLM-Index and arousal index in age-, sex-, and BMI-matched obese individuals with moderate and severe OSA. Taken together, higher AHI was accompanied by a disturbed sleep architecture at large. Of interest, TST and the distribution of sleep stages did not differ significantly between the two groups. In addition, there was no significant difference in CRP serum levels between obese individuals with mild OSA compared to those in an age-, sex-, and BMI-matched group with moderate/severe OSA. Most importantly, the serum levels of HbA1c were significantly higher in obese individuals with moderate to severe OSA compared to age-, sex-, and BMI-matched obese individuals with mild OSA.

It is well-known that obesity is one of the main predisposing factors for OSA and studies have demonstrated a clear correlation between obesity and the development of sleep-disordered breathing [2,5,15]. It has been suggested previously that obesity worsens OSA through targeted fat deposition in the tissues surrounding the upper airway, resulting in decreased upper airway lumen and increased upper airway collapsibility [16,17]. Conversely, not all obese individuals have OSA. Although there is a general consensus on the need for OSA screening in obese individuals and other associated disorders [15], less than 10% of individuals with OSA are diagnosed and/or treated, therefore increasing the risk for comorbidities and all-cause mortality from OSA in the general population [18]. The metabolic syndrome is among the most common OSA comorbidities. However, the role of OSA in the pathogenesis of insulin resistance is not fully understood [19,20]. The hypothesis that OSA may represent an independent risk factor for the development of T2DM has been supported by a recent meta-analysis of prospective cohort studies [19]. The relationship between OSA and glycemic parameters in obese non-diabetic individuals is poorly defined. Cignarelli et al. showed HbA1c and AHI to be significantly correlated in obese subjects in a randomized controlled trial. The authors suggested that metabolic abnormalities may be linked not only to obesity but also to concomitant OSA [21]. Another study by Kent et al. found that, in non-obese individuals, HbA1c levels independently correlated with the AHI values. The authors concluded that OSA severity is independently predicative of glycemic health [22]. The findings of the aforementioned studies support the results of the present study. Accordingly, in our study, HbA1c levels were significantly higher in obese individuals with AHI ≥ 15/h. This core finding supports the hypothesis that AHI, and possibly other OSA-related respiratory parameters, may be of outstanding importance in the regulation of glycemic control, particularly in obese individuals. This argument may also involve non-obese individuals; nonetheless, this was not a subject of investigation in the present study. Accordingly, Tasbakan et al. provided evidence for a dose–response relationship between OSA treatment during long-term positive airway pressure (PAP) therapy and improved glycemic control in morbidly obese patients [23]. In their study, HbA1c was significantly reduced by PAP therapy, so the authors stressed the importance of PAP therapy to substantially modify diabetic complications in OSA patients.

Serum CRP has attracted much interest as a significant biomarker of systemic inflammation in OSA. CRP is regarded as a significant serum marker of inflammation. Several studies testified that CRP is a vital factor in cardiovascular diseases, such as atherosclerosis, myocardial infarction, and stroke in OSA [24]. A recently published meta-analysis suggested CRP levels to be significantly elevated in patients with OSA, particularly when BMI was more than 30 kg/m^2^ and OSA severity was moderate to severe. Due to its inflammatory pathophysiology, the authors supported the role of CRP as a pivotal biomarker in OSA [25]. In our study, although CRP levels tended to be higher in individuals with moderate and severe OSA compared to those with mild OSA, there was no statistically significant difference between the two age-, sex-, and BMI-matched groups. The results of various studies on the issue are inconsistent. Kanbay et al. showed CRP levels to be significantly higher in OSA patients compared to healthy controls [26]. However, Guilleminault et al. found no correlation between CRP levels and OSA severity. The authors concluded that only BMI was associated with elevated CRP levels [27]. Notably, we took special care to ensure an equal distribution of female participants in both groups (i.e., exactly 70% in both groups) because we are aware of the differential effect of sleep-disordered breathing on serum CRP levels between sexes [28]. Gouveris et al. found sleep-disordered breathing to be more strongly associated with systemic inflammation in females than in males [28]. In addition, Rocchi et al. proved that CRP was elevated in both males and females, with an ascending trend from males with moderate OSA to females with severe OSA [29]. The authors suggested OSA has a strong link with cardiovascular and cerebrovascular morbidity, and it is worth considering that this might be influenced by patients’ sex [29]. In a recently published study by Buratti et al., men showed more severe polygraphic parameters and an increased low-to-high frequency ratio as an index for the sympathovagal balance compared to women [30]. However,, the link between elevated CRP levels and OSA severity remains controversial. Both parameters (systemic inflammation and OSA) may potentiate the risk for cardiovascular morbidity. Fleming et al. found that elevated CRP or HbA1c serum levels, and particularly their concomitant elevation, may be quite strong biomarker-based predictors of OSA. The authors proposed the use of these two biomarkers or OSA screening by primary care providers, particularly in high-risk populations [18].

Our study has certain limitations. A major limitation of our study is the relatively small sample size. Nonetheless, although the results obtained in our study come from retrospective data, we were able to reach a very successful age-, sex-, and BMI-matching between the two study groups. A potential confounding factor is that serum levels of CRP and HbA1c may have been influenced by dietary control or recent changes in lifestyle in selected individuals, which could not be captured by a retrospective data analysisHowever, acute or chronic-potentially confounding-inflammatory conditions were excluded by means of historical information found in the patients’ charts. Despite careful review of all medical files, due to the retrospective nature of this study, it cannot be guaranteed that all chronic diseases or recent/acute inflammatory states were reported by the patients when their histories were obtained.

The lack of capillary blood gas analysis is another important limitation of our study. Capillary blood gas analysis enables the diagnosis of the obesity hypoventilation syndrome (OHS), which, in turn, can contribute to pulmonary hypertension, independently increasing serum levels of CRP [31].

To the best of our knowledge, this is the first study that was carried out to compare different clinical characteristics in obese age-, sex-, and BMI-matched individuals with mild OSA to those with moderate to severe OSA. Although we were able to demonstrate a clear positive association between the OSA respiratory severity and HbA1c levels, as well as a respective trend regarding CRP levels, an explanation for the possible link between OSA and the development of diabetic or cardiovascular conditions remains unclear. The latter issue should be a matter of future, prospective studies with larger sample sizes and a detailed focus on associated molecular and cellular mechanisms. The timely diagnosis and treatment of OSA in obese individuals may play a significant clinical role in the prevention of diabetic conditions.

## 5. Conclusions

OSA severity is associated with elevated HbA1c levels in obese age-, sex-, and BMI-matched individuals. CRP, as a biomarker of systemic inflammation, may not be significantly affected by OSA severity, but tends to be elevated in obese patients. Therefore, OSA respiratory severity appears to be a major factor associated with the disruption of glycemic control in obese individuals.

## Figures and Tables

**Table 1:** Baseline characteristics.

	AHI < 15	AHI ≥ 15	Between Group Comparison (*p*-Value)
Number of individuals	23	23	
Male participants (%)	7 (30)	7 (30)	
Female participants (%)	16 (70)	16 (70)	
Age, in years (± SD)	42.57 ± 11.44	43.43 ± 11.96	0.214
BMI, in kg/m^2^ (± SD)	37.35 ± 3.88	37.87 ± 4.74	0.532
Hypertension (n (%))	10 (43)	8 (35)	
Diabetes mellitus (n (%))	2 (9)	2 (9)	
Insulin intake (n(%))	0 (0)	0 (0)	
Metformin intake (n (%))	3 (13)	2 (9)	
Pulmonary disorders (n (%))	2 (9)	2 (9)	
Hypothyroidism (n (%))	4 (17)	6 (25)	
Levothyroxine intake (n (%))	5 (22)	6 (25)	
Number of non-smokers (%)	16 (70)	15 (65)	
Number of active smokers (%)	7 (30)	8 (35)	

BMI-body mass index. Note: In both groups, there were two diabetics taking Metformin. One additional patient in group “AHI < 15” was taking Metformin for dietary reasons but did not have diabetes.

**Table 2:** Sleep parameters in obese individuals with mild or moderate to severe obstructive sleep apnea.

	AHI < 15	AHI ≥ 15	Between Group Comparison (*p*-Value)
AHI (n/hour)	7.5 (5.5–12.5)	41.5 (25.7–71.8)	<0.001
Apnea index (n/hour)	1 (0.3–2.5)	11.3 (5.7–35.1)	<0.001
Hypopnea index (n/hour)	6.2 (4.3–8.7)	23.2 (17–29.6)	<0.001
Snoring index (n/hour)	114 (10–350)	425.2 (233–700.6)	0.010
Oxygen desaturation index (n/hour)	9 (5.7–13)	40.9 (25.5–72.7)	<0.001
Oxygen desaturation in REM (n)	16 (6–25)	49 (24–78)	<0.001
Average oxygen desaturation (%)	95 (94–95)	92 (91–94)	<0.001
t90 (%)	0.3 (0.1–1.9)	8.4 (3–22.6)	<0.001
PLM (n)	19 (4–40)	128 (24–211)	0.014
TST (min)	387.12 ± 41.39	369.85 ± 43.18	0.312
N3 sleep (%)	19.99 ± 9.82	16.1 ± 9.13	0.39
REM sleep (%)	15.23 ± 7.62	13.73 ± 6.38	0.746
REM AHI (n/hour)	17.6 (12.8–30.4)	56 (40–78)	<0.001
Arousal index (n/hour)	10.2 (8.5–11.8)	23.6 (13.7–33.8)	<0.001
Respiratory arousal index (n/hour)	3.9 (2.6–7.7)	13.3 (10.5–21.8)	<0.001
AHI in supine position (n/hour)	10.1 (6.1–22)	62.7 (35.6–79)	<0.001

AHI—apnea–hypopnea index; REM—rapid eye movement; t90—percentage of oxygen desaturation lower than 90%; PLM—periodic limb movement; TST—total sleep time; REM AHI-AHI during REM sleep. Data are presented as mean ± standard deviation for normal distributed or median and interquartile range (IQR) for non-normal distributed values.

**Table 3:** C-reactive protein and glycated hemoglobin levels in obese individuals with mild or moderate to severe obstructive sleep apnea.

	AHI < 15	AHI ≥ 15	Between Group Comparison (*p*-Value)
CRP (mg/L)	4.25 (2.25–7.2)	7.8 (3.75–9.7)	0.186
HbA1c (%)	5.2 (5–5.75)	5.8 (5.35–6.85)	0.016

CRP-C—reactive protein. HbA1c—glycated hemoglobin. The non-normal distributed values are presented as median and interquartile range (IQR).

## Data Availability

The data presented and analyzed in this study are available on reasonable request by the corresponding author.
